# Clinical presentation vs endoscopy for an early diagnosis of eosinophilic esophagitis: a case report

**DOI:** 10.17179/excli2022-4838

**Published:** 2022-03-24

**Authors:** Michele Di Stefano, Giacomo Grandi, Vera Bonaso, Elisabetta Pagani, Gino Roberto Corazza, Antonio Di Sabatino

**Affiliations:** 1 1st Department of Internal Medicine, IRCCS "S. Matteo" Hospital Foundation, Pavia, Italy; 2 University of Pavia, Italy

**Keywords:** eosinophilic esophagitis, esophageal eosinophilia, dysphagia, esophageal dysphagia

## Abstract

Eosinophilic esophagitis (EoE) is a type-2 mediated, chronic inflammatory disease showing an increase of both incidence and prevalence. Early diagnosis is mandatory, to prevent fibrostenotic complication of the disease. Due to the low sensitivity of the classic endoscopic features of the disease, a strong clinical suspicion should drive the decision to collect mucosal biopsies of the esophagus. We describe the case of an atopic patient suffering from dysphagia with normal esophageal mucosa and frank histological hallmarks of the disease.

## Introduction

Eosinophilic esophagitis (EoE) is a type 2 chronic inflammatory disease, characterized by mucosal eosinophilic infiltrate evoking symptoms of esophageal dysfunction which can lead to sequelae of fibrosis and strictures (Furuta and Katzka, 2015[7]). The pathophysiology of this condition is largely unknown, but a strict correlation with atopic comorbidities is evident (Furuta and Katzka, 2015[7]): food antigens are considered the main actors, even if aeroallergens seem to have a role in a subgroup of patients (Van Rhijn et al., 2013[14]). Both incidence and prevalence of EoE showed an impressive increase in the last 30 years (Dellon and Hirano, 2018[4]), independently from the increase of disease awareness or increase of the number of esophageal biopsies (Dellon et al., 2015[2]). Accordingly, more attention should be reserved to EoE diagnosis. On clinical grounds, dysphagia and reflux-like symptoms are considered the most common clinical manifestation of the disease (Dellon et al., 2009[3]) and food impaction is considered a pathognomonic sign (Alexander et al., 2019[1]). The occurrence of this latter sign is associated to the endoscopic detection of typical mucosal aspects, consisting of circumferential rings, linear furrows, whitish papules suggesting eosinophilic microabscesses, esophageal strictures or reduction of esophageal lumen dimension (Dellon et al., 2009[3]; Hirano et al., 2013[8]). Endoscopic signs are a guide to perform biopsies in the esophagus to detect the histologic hallmark of EoE (Dellon et al., 2009[3]). However, the case we report shows the presence of histologic signs of the disease before the appearance of endoscopic signs, thus suggesting to modify the diagnostic algorithm for this condition in order to reduce the diagnostic delay and improve therapeutic approach. 

## Case Report

In 2013, a 52-year-old female presented to the gastroenterology outpatient clinic with dysphagia for both solids and liquids. She reported since 1998 some severe episodes of dysphagia with an unpleasant feeling of bolus transit stop at the level of the mid esophagus associated to sialorrhoea, spontaneously reverted without the need for a therapeutic endoscopy. Comorbidities included carotid atherosclerosis on ASA, homozygosis for MTHFR gene mutation and atopy with prick test positivity for sensibilization to cat epithelium with oculorhinitis manifestations but not bronchospastic reactions. At the beginning of symptoms, in 1998, upper endoscopy showed regular esophageal canalization, a small hiatal hernia, the absence of esophageal and duodenal mucosa lesions and the presence of a juxtapyloric mucosal erosion. Mucosal biopsies for standard histological evaluation were not collected. It was performed only a rapid test for *Helicobacter pylori*, which resulted positive and the patient underwent eradication treatment with success. Thereafter, some courses of PPI were prescribed to treat symptom relapse. 

Abdominal and thoracic examination were unremarkable. Routine laboratory tests were normal. Videofluoroscopic swallowing study and esophageal transit evaluation did not reveal the presence of structural or functional alterations: in particular, the transit of liquid, semi-solid and solid boluses were normal. Esophageal manometry detected normal progression of the peristaltic waves, but wave amplitude was much increased (> 300 mmHg) together with a mild hypertensive LES (32 mmHg). 

She underwent upper endoscopy showing normal esophageal, gastric and duodenal mucosa. Gastric and duodenal histology were unremarkable but a significant esophageal infiltrate at distal, medium and proximal esophagus, with eosinophilic counts of 169, 58 and 29 cells per high-power field, respectively, was shown.

A diagnosis of EoE was done and prednisone 25 mg/day (0.5 mg/kg body weight) per 2 weeks followed by a gradual tapering in 6 weeks was prescribed. The patient experienced a complete clinical remission for 2 years. Due to the occurrence of reflux-related symptoms, in particular moderate heartburn and mild regurgitation, two courses of 4-week PPI were prescribed. 

**Figure 1:**
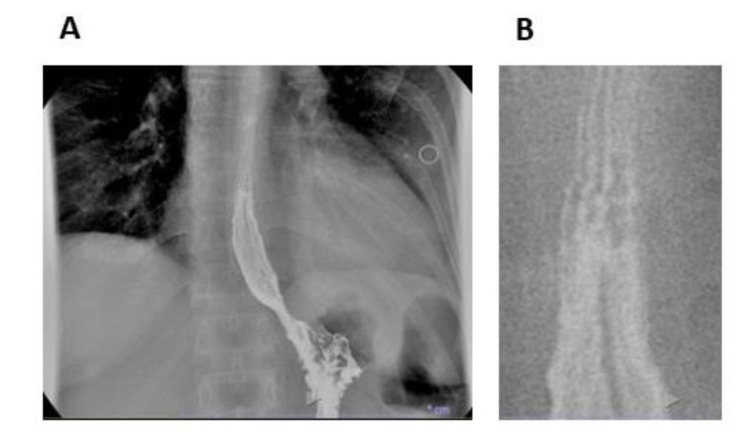
Videofluoroscopic swallowing study and esophageal transit evaluation. (A) Alterations of esophageal profile. (B) Detail of the proximal portion of barium column in A. Esophageal mucosa contrasted with a low amount of barium shows some horizontal lines suggesting the presence of rings.

**Figure 2:**
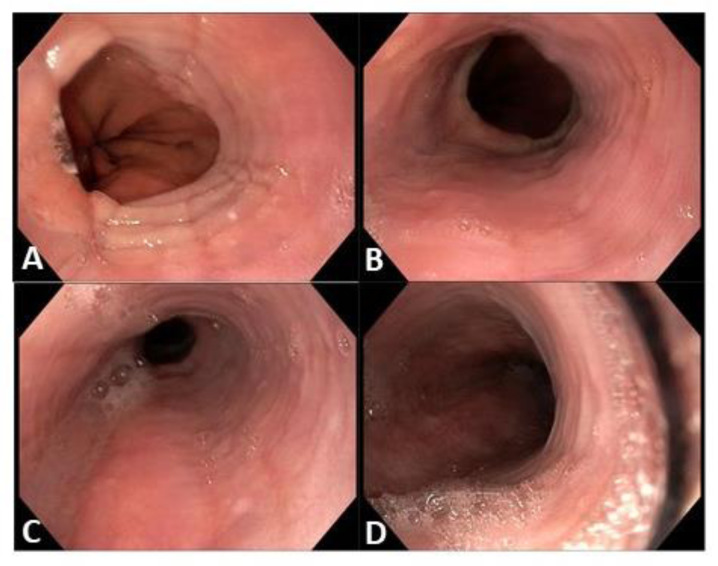
Endoscopic appearance of esophageal mucosa. (A) Rings and furrows at the distal esophagus, the juxtacardiac tract. (B) Rings at the distal esophagus. (C) Rings, furrows and exudates at the mid esophagus. (D) rings at the proximal esophagus

In 2018, she experienced a flare of symptoms. A videofluoroscopic swallowing study and esophageal transit evaluation was repeated. The presence of mildly spiculed esophageal profile was evident (Figure 1(Fig. 1)). Upper endoscopy showed mucosal rings, furrows and exudates along the esophagus (Figure 2(Fig. 2)). Esophageal histology confirmed the presence of a significant intraepithelial eosinophilic infiltrate, with eosinophilic count of 60/HPF. Accordingly, the patient begun a 12-week period of budesonide 9 mg/die associated to pantoprazole 40 mg/die, tapered to budesonide 6 mg/die associated to pantoprazole 40 mg/die for further 12 weeks. The complete recovery of symptoms induced the reduction of budesonide dosage at 3 mg/day for another 12-week period. To minimize side effects, a maintaining treatment with 12-week period of budesonide alternated with 12-week period of PPI was prescribed and still ongoing, with complete absence of dysphagia and resolution of endoscopic esophageal signs. 

## Discussion

The diagnosis of EoE is based on the detection of a significant eosinophilic infiltrate in esophageal mucosa, but mucosal sampling in esophagus does not represent a commonly performed procedure during routine endoscopic practice. Consequently, esophageal conditions characterized by histological lesions and macroscopically normal mucosa are diagnosed only if a strong clinical suspicion is present, determining the need for biopsies. Accordingly, a consensus on biopsy protocol in these conditions is essential. 

Endoscopic detection of typical esophageal mucosal lesions, suggesting EoE diagnosis, represents the indication for mucosal sampling: when esophageal signs are present, histological pathognomonic signs of EoE are generally also present. However, the reported case shows that eosinophilic infiltrate may be present before endoscopic signs develop and this is a very important issue as suggests that the best practice allowing an early diagnosis of EoE improving the diagnostic delay should be based on a modification of the indications for mucosal sampling. 

It should be considered that natural history of EoE is characterized by a progression of inflammation to fibrosis (Dellon and Hirano, 2018[4]). It was shown that patients with inflammatory phenotype are younger than patients with mixed or fibrostenotic phenotype, the risk of developing a fibrostenotic phenotype doubles for every decade of life and, in symptomatic patients, each year of diagnostic delay results in an increase of 5 % of risk of developing a fibrostenotic phenotype (Dellon et al., 2014[5]). In particular, in patients with a diagnostic delay less than 2 years, the prevalence of fibrotic signs is 46.5 %, but in patients with a diagnostic delay higher than 20 years, the prevalence of fibrotic signs is 87.5 %. Considering that mean diagnostic delay in EoE is 6 years (interquartile range 2-12 years) (Schoepfer et al., 2013[12]), it could be calculated that, in adult patients, at diagnosis, a risk of fibrostenotic phenotype higher than 60 % is already present. Pediatric patients show an inflammatory phenotype but the rapid evolution trough fibrostenosis in adult patients and the entity of the mean diagnostic delay suggest that in adults the therapeutic window during which dietary and pharmacological treatment are effective is very short. 

Consequently, a different strategy for the collection of mucosal biopsies should be adopted and, by giving value to the association of atopy and dysphagia in young adults, an improvement in the early diagnosis of EoE could be achieved. Accordingly, in subjects with upper gastrointestinal symptoms, when dysphagia is present, it should be also revised the indication to upper endoscopy, as a recent revision of guidelines aimed at the prevention of gastric cancer suggested an increase of the age for upper endoscopy from 55 (Talley et al., 2005[13]) to 60 years (Moayyedi et al., 2017[11]). It was previously shown in Denmark that a more aggressive modification of biopsy protocol in patients with dysphagia, regardless of the macroscopic appearance of the esophagus, resulted in a 50-fold increase of histological diagnosis of esophageal eosinophilia (Krarup et al., 2021[10]). Moreover, it was recently described that 11.5 % of patients with symptomatic EoE may show endoscopically normal esophagus (Eluri et al., 2021[6]): in comparison with patients with a diagnostic endoscopy, the subgroup of EoE patients with macroscopically normal esophagus showed a pediatric age, a shorter diagnostic delay, a higher prevalence of abdominal pain and a low prevalence of dysphagia and food impaction, both characteristics of pediatric age. 

The introduction in 2013 of Endoscopic Reference Score (EREFS) classification system for endoscopic assessment of EoE associated esophageal lesions significantly decreased the number of EoE patients with normal esophagus, suggesting that the availability of a classification system increased the awareness of endoscopists about this conditions. Consequently, more esophageal biopsies were performed and more diagnosis were made (Eluri et al., 2021[6]). To tell the truth, the need for a more aggressive approach was already stressed in 2012, before the introduction of an EREFS classification system by Kim and coworkers (2012[9]): in a meta-analysis, these authors showed endoscopic signs are characterized by a high specificity, ranging from 90 to 95 %, but a very low sensitivity ranging from 15 to 48 %. They concluded that when EoE is strongly suspected on clinical grounds, biopsy specimens should be obtained regardless of macroscopic appearance of the esophagus. 

In conclusion, the case we described strengthens the evidence that the suspect of EoE is based on clinical characteristics of patients: we need to give more value to clinical evidence and less value to endoscopic signs. Therefore, when EoE is suspected, according to the very low negative predictive value of a normal esophageal mucosa and the increase of EoE diagnosis with a more aggressive approach, biopsy samples must be always collected, even when esophageal mucosa is normal. 

## Conflict of interest

The authors declare that they have no conflict of interest.
